# Intraocular Inflammation Following Intravitreal Faricimab Injection in Neovascular Age-Related Macular Degeneration

**DOI:** 10.7759/cureus.75937

**Published:** 2024-12-18

**Authors:** Shweta Parakh, Vaibhav Bhatt, Shrutanjoy Das, Priksha Lakhlan, Gaurav Luthra, Saurabh Luthra

**Affiliations:** 1 Ophthalmology, Drishti Eye Institute, Dehradun, IND

**Keywords:** brolucizumab, faricimab, intraocular inflammation, uveitis, vascular occlusion, vasculitis, vitritis

## Abstract

We herein report intraocular inflammation (IOI) following intravitreal (IVT) faricimab injection in three patients. A 73-year-old male, a 68-year-old female, and an 82-year-old female, all diagnosed with neovascular age-related macular degeneration (AMD), had received multiple anti-vascular endothelial growth factor (anti-VEGF) injections for the same. They were injected with IVT faricimab due to non-response to other agents.

Following IVT faricimab, two patients presented with mild vitritis (grade 2) within three days post-injection, and one patient presented with anterior uveitis on day 1. No associated retinal vasculitis or occlusion was present. All patients responded well to topical steroid therapy. Notably, the 68-year-old lady had a history of IOI (mild vitritis) following a single IVT brolucizumab in the past. The other two patients had not received IVT brolucizumab earlier.

IOI after IVT faricimab has been reported a few times previously. One must be aware of the spectrum of presentation (vitritis, anterior uveitis, panuveitis, vascular occlusion, vasculitis) and be vigilant to provide timely management, especially in sight-threatening cases. A history of IOI following previous brolucizumab injection needs to be evaluated further for possible immunological mechanisms at play.

## Introduction

Faricimab is an FDA-approved novel bispecific, monoclonal antibody that targets both vascular endothelial growth factor-A (VEGF-A) and angiopoietin-2 (Ang-2) implicated in neovascularization and vascular leakage [1]. A more complete blockage of neovascularization and exudation with improved vascular stability is possible due to the simultaneous inhibition of both these pathways by the bispecific agent [2]. Faricimab has been approved in the US for treating neovascular age-related macular degeneration (nAMD) (based on the TENAYA and LUCERNE trials), diabetic macular edema (DME) (from the YOSEMITE and RHINE trials) since 2022, and retinal vascular occlusion (RVO) in 2023 (supported by the BALATON and COMINO trials) [1].

Reports of intraocular inflammation (IOI) are uncommon with faricimab. Reported rates of IOI (excluding endophthalmitis) were statistically higher than the aflibercept groups as follows: 17 of 1262 in DME (1.3%), 13 of 664 cases in nAMD (2.0%), and 9 of 641 in RVO (1.4%) [1]. In comparison, reported rates of IOI among other intravitreal agents include pegcetacoplan 2.1%-3.8% [3], brolucizumab 4.6% [4], ranibizumab 0.005-0.02% [5], aflibercept 0-0.16% [5], and bevacizumab 0.081-0.10% [5].

Herein, we report a series of three cases of IOI (two with vitritis without retinal vasculitis and one with anterior uveitis) associated with IVT faricimab and provide a literature review for this infrequently reported complication.

## Case presentation

Case 1

**Figure 1:**
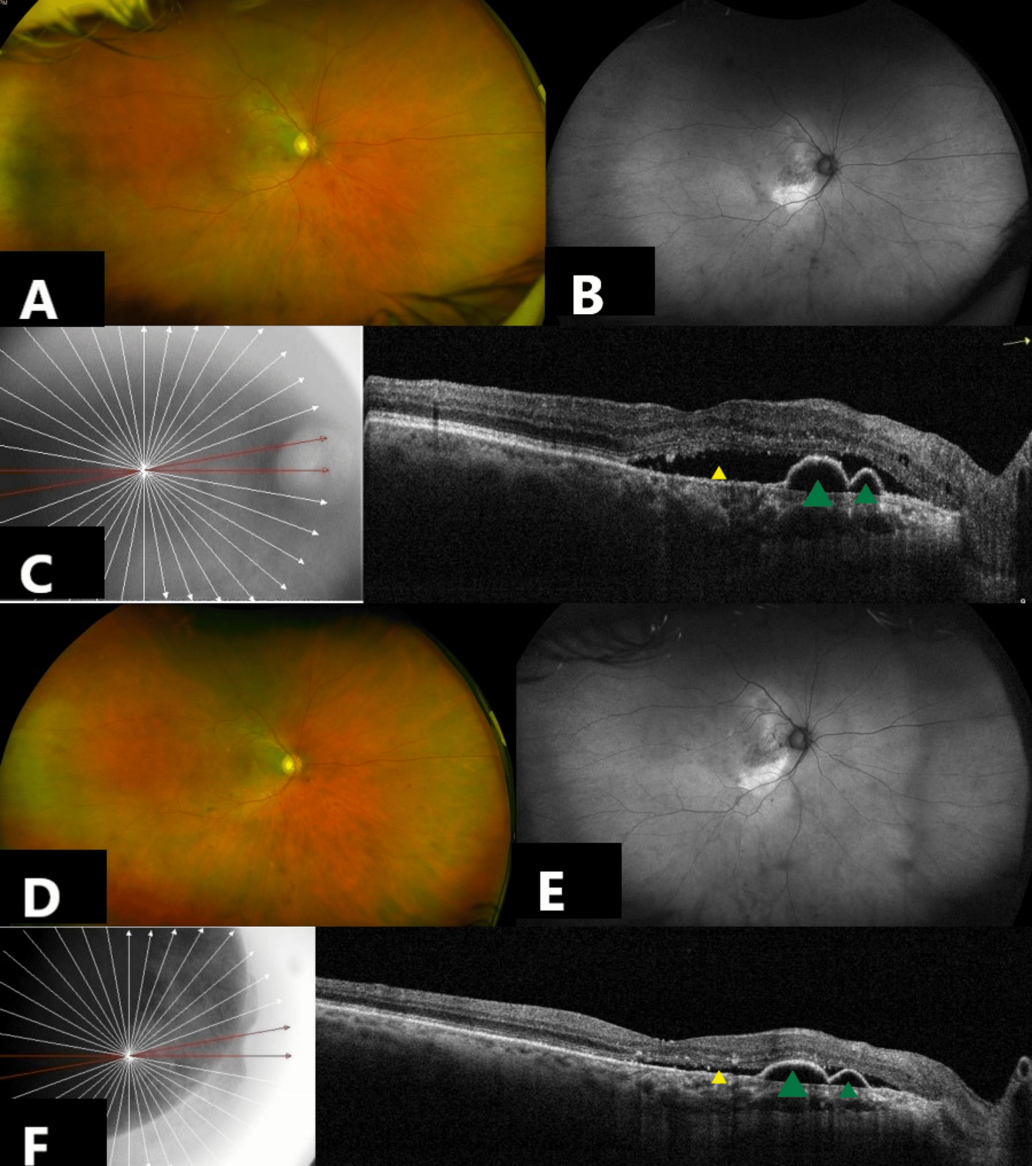
Multimodal Imaging of a 73-year-old Male with idiopathic polypoidal choroidal vasculopathy (IPCV) and diabetic retinopathy. A, B: Ultra-widefield fundus photographs of the right eye (OD) at baseline (A-color, B-autofluorescence), showing moderate non-proliferative diabetic retinopathy (NPDR) and perifoveal retinal pigment epithelium (RPE) alterations in the right eye. C: Spectral-domain optical coherence tomography (SD-OCT) of the right eye at baseline, showing subretinal fluid (SRF) (yellow arrowhead) and a notched pigment epithelial detachment (PED) (green arrowheads). D, E: Ultra-widefield fundus photographs of the right eye at seven days post-intravitreal faricimab injection, showing stable findings with no evidence of vasculitis or vascular occlusion. Mild vitritis (1+) was observed and managed with topical treatment. F: SD-OCT of the right eye one-month post-injection, demonstrating a reduction in SRF (yellow arrowheads) and mild flattening of the PED (green arrowheads).

A 73-year-old male, diagnosed with bilateral oculus uterque (OU) idiopathic polypoidal choroidal vasculopathy (IPCV) and moderate non-proliferative diabetic retinopathy (NPDR), had been under regular follow-up for the past five years in the retina clinic. He was a known diabetic (controlled on oral hypoglycemic agents and insulin) and a well-controlled hypertensive patient. Past surgical history was relevant for pseudophakia OU. He had received ten intravitreal (IVT) aflibercept injections in the past in his right eye, oculus dexter (OD). The decision to switch from IVT aflibercept to IVT faricimab was made in view of persistent pigment epithelial detachment (PED) and subretinal fluid (SRF) in non-response to aflibercept. He received one dose of IVT faricimab OD. Pre-injection best corrected visual acuity (BCVA) was 6/18 OD and 6/9 oculus sinister (OS). The anterior segment was quite OU. No vitreous cells were noted OU. Fundus examination and ultra-widefield imaging (Optos PLC, Dunfermline, United Kingdom) revealed moderate non-proliferative diabetic retinopathy (NPDR) and perifoveal retinal pigment epithelium (RPE), and alteration in OD (Figure 1A, Figure 1B). Similar fundus findings were seen in OS. Spectral-domain optical coherence tomography (SD-OCT) (RTVue XR Avanti, Optovue Inc., Fremont, CA, USA) OD showed subretinal fluid (SRF) and a notched pigment epithelial detachment (PED) (Figure 1C). Pre-injection central macular thickness (CMT) was 337 microns OD. The left eye (OS) was closely monitored for an extrafoveal PED and minimal SRF with CMT 275 microns.

On the seventh day post IVT faricimab, best-corrected visual acuity (BCVA) was stable at 6/18 OD. IOP was 11 mm Hg OD. The anterior chamber was quiet OD. Vitreous cells 1+ were noted in OD. Fundus examination was stable OD (Figure 1D, Figure 1E). There was no evidence of vasculitis or vascular occlusion, as documented on ultra-widefield imaging. This mild vitritis responded well to treatment with topical prednisolone acetate 1% eyedrops, tapered gradually over the next four weeks. One month post-injection, SD-OCT showed a reduction in SRF and mild flattening of PED OD (Figure 1F). Post-injection CMT was 293 microns OD. BCVA was maintained at 6/18 OD. Fundus examination remained stable OD. The patient continues to be under close follow-up.

Case 2

A 68-year-old lady diagnosed with dry age-related macular degeneration (AMD) OD and wet AMD OS had been under treatment and follow-up at our clinic since 2018. During her treatment for a scarring choroidal neovascular membrane (CNVM) OS, she received four IVT ranibizumab and 12 IVT aflibercept injections. She did not show any regression of fluid after the last two aflibercept injections and was therefore switched to IVT brolucizumab. Although intraretinal fluid resolved in response to IVT brolucizumab, the patient had asymptomatic mild vitritis one week following the same. This responded well to treatment with topical prednisolone acetate eyedrops 1%, tapered gradually over four weeks. Subsequently, she was switched back to IVT aflibercept in view of mild IOI after IVT brolucizumab. She continued to have a suboptimal response to four more aflibercept injections and was then switched to IVT faricimab (as soon as it was available).

**Figure 2:**
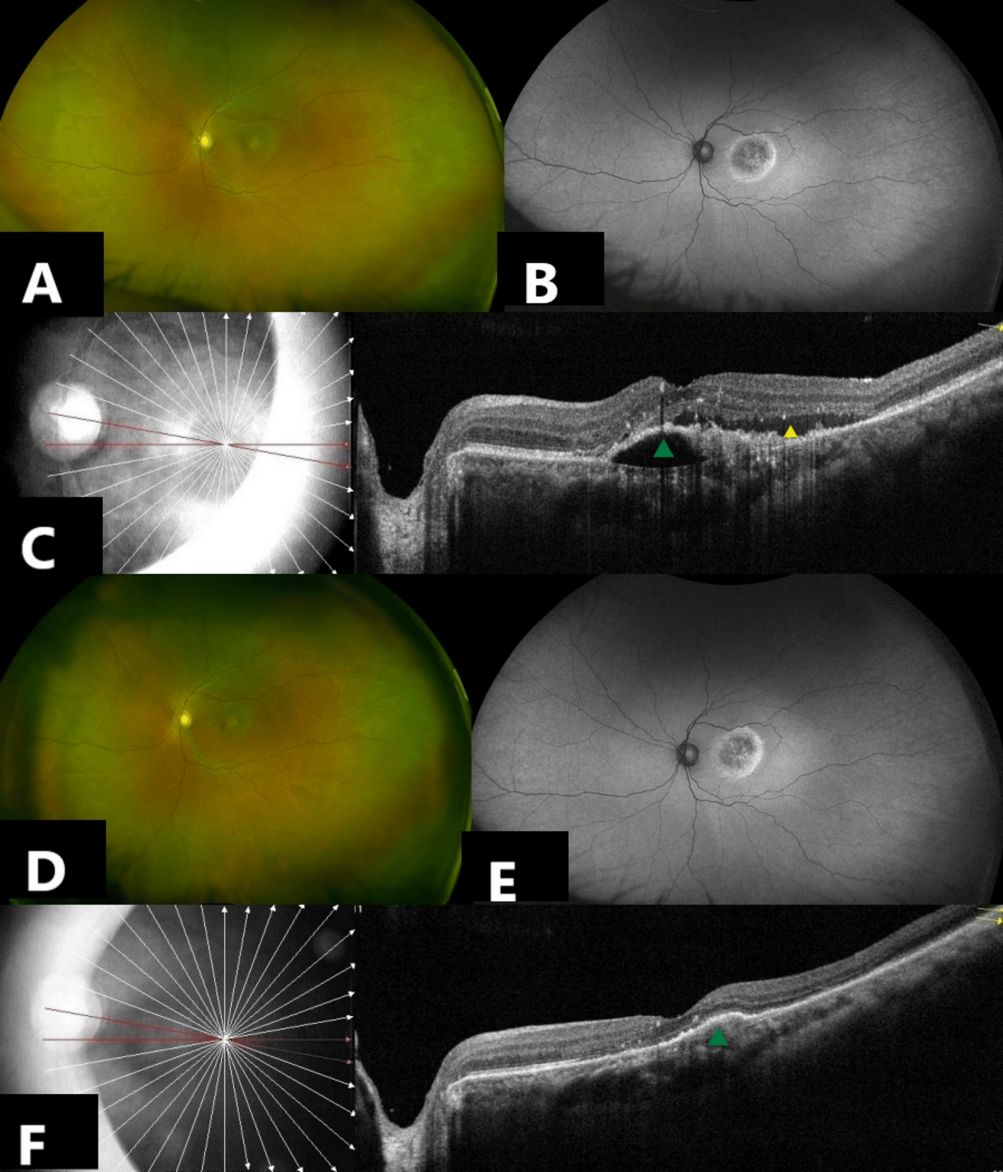
Multimodal Imaging findings of a 68-year-old Female with neovascular age-related macular degeneration (nAMD) and suboptimal response to multiple anti-VEGF therapies. A, B: Ultra-widefield fundus photographs of the left eye (OS) at baseline (color and autofluorescence) showing scarring CNVM OS. C: Spectral-domain optical coherence tomography (SD-OCT) of the left eye (OS) at baseline showing choroidal neovascular membrane (CNVM) with subretinal fluid (SRF) (yellow arrowhead) and irregular pigment epithelial detachment (PED) (green arrowhead). D, E: Ultra-widefield  fundus photograph OS  one week post-intravitreal faricimab injection, showing stable findings with no evidence of vasculitis or vascular occlusion. F: SD-OCT OS showing resolution of SRF and flattening of PED (green arrowhead) at one month post-injection faricimab.

Prior to receiving IVT faricimab, BCVA was 6/6 OD and 6/18 OS. IOP was 12 and 13 mmHg, respectively, in OD and OS. A slit lamp examination showed a quiet anterior chamber and pseudophakia OU. No vitreous cells were noted OU. Fundus examination showed drusen at macula OD. Ultra-widefield imaging showed a scarring CNVM OS (Figure 2A, Figure 2B). SD-OCT showed CNVM with SRF and irregular PED OS (Figure 2C). Pre-injection central macular thickness (CMT) was 424 microns OS. One week post-IVT faricimab OS, BCVA improved to 6/12p OS. The anterior chamber (AC) was quiet OS. Vitreous cells 1+ were noted in OS. Fundus examination did not show any vasculitis or vascular occlusion (Figure 2D, Figure 2E). Topical steroid therapy (prednisolone acetate 1% eyedrops QID) was started in a weekly tapering regimen for four weeks. Vitreous cells had resolved by the second week of this regimen. At a one-month follow-up visit, BCVA improved to 6/12 OS. The AC was quiet, and no vitreous cells were present in OS. SD-OCT showed resolution of SRF and flattening of PED OS (Figure 2F). Post-injection CMT was 199 microns OS.

Case 3

**Figure 3:**
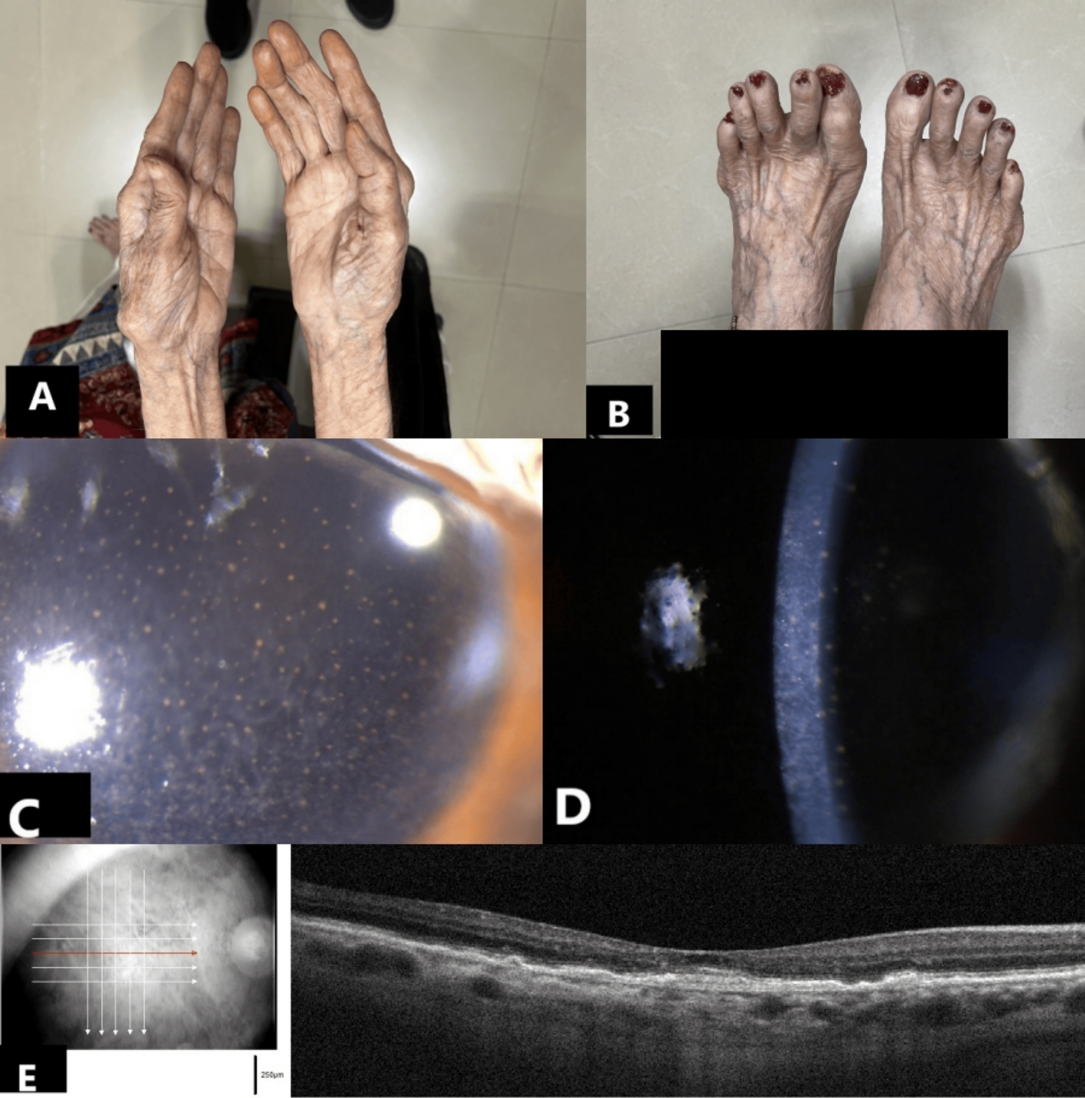
Clinical findings in an 82-year-old female with wet age-related macular degeneration (AMD) in the right eye (OD) who was switched to intravitreal faricimab after non-response to intravitreal aflibercept. (A, B) Systemic examination showing evidence of senile osteoarthritis, with negative results for rheumatoid factor and anti-CCP antibodies. (C, D) Slit lamp photographs showing multiple pigmented keratic precipitates (KPs) in the right eye one day post-injection faricimab. (E) OCT macula showing scarring choroidal neovascular membrane (CNVM) in the right eye with resolution of subretinal fluid (SRF) at 1-month follow-up.

An 82-year-old female with OD wet AMD and OS dry AMD was switched to IVT faricimab in view of non-response to four doses of IVT aflibercept. Systemic examination revealed senile osteoarthritis (Figure 3A, Figure 3B), which was negative for rheumatoid factor and anti-cyclic citrullinated peptide (anti-CCP) antibodies. Prior to receiving IVT faricimab, BCVA was 6/18 OD and 6/9 OS. IOP was 14 mm Hg OU. Both eyes were quiet with pseudophakia.

On day one following IVT faricimab OD, she presented with blurred vision in the same eye. BCVA was 6/24 OD. IOP was 15 mm Hg OD. Slit lamp examination showed multiple pigmented keratic precipitates (KPs) (Figure 3C, Figure 3D) and mild flare OD. There was no evidence of vasculitis or vascular occlusion. Rigorous topical steroid therapy (prednisolone acetate 1% eyedrops Q2h) was started in a weekly tapering regimen for four weeks. KPs had resolved by the second week of the regimen. At a one-month follow-up visit, BCVA improved to 6/18 OD. The anterior chamber was quiet, and no vitreous cells were present OD. OCT macula showed scarring CNVM with a resolution of SRF (Figure 3E).

## Discussion

Recent reports of IOI following faricimab include one series of three patients from a single institute with initially culture-negative severe IOI [6]. All three patients received intravitreal ceftazidime, 2.2 mg/0.1 mL, and vancomycin, 1 mg/0.1 mL, immediately following vitreous taps. All vitreous tap culture results were negative. One patient underwent vitrectomy one-day following presentation and reported growth of one colony of *Staphylococcus epidermidis* in the vitreous culture. This was adjudged a likely contaminant by infectious disease specialists. Notably, the injections belonged to two different lot numbers administered at three locations in the same institute between September 20 and October 20, 2023.

In a prospective case study of 71 eyes of 71 patients whose treatment was switched to faricimab from other anti-vascular endothelial growth factors (VEGF) agents for neovascular age-related macular degeneration (nAMD) and polypoidal choroidal vasculopathy (PCV), one patient showed IOI in the form of mild vitritis without vasculitis [7]. This was resolved with topical steroids and no sequelae. Li et al. reported a case of bilateral occlusive vasculitis associated with bilateral intravitreal faricimab injections that were treated with a seven-week tapering course of oral and topical corticosteroids [8]. Two cases of granulomatous uveitis with elevated intraocular pressure (IOP) have been described following IVT faricimab by Palmieri et al. [9].

Sterile IOI can present in two forms: a severe acute-onset inflammation, often mimicking infectious endophthalmitis or a delayed-onset inflammation with or without occlusive retinal vasculitis. The latter has been associated with IVT brolucizumab [10] and pegcetacoplan [3]. Most often, patients present with floaters about 10-14 days post-injection.

Sterile intraocular inflammation following intravitreal anti-VEGF injections can be attributed to several potential patient-related, drug-related, or manufacturing-related factors. Patient-related factors that may lead to increased susceptibility to immunologic reactions include a history of uveitis, the use of pro-inflammatory prostaglandins, or a history of diabetes and AMD, which may affect the blood-retinal barrier [11]. The presence of treatment-boosted antidrug antibodies (ADAs) was found to be approximately 44% for brolucizumab, which could potentially contribute to the increased IOI incidence following IVT brolucizumab [10].

The study of the drug molecule provides further insight into its role in inciting inflammation. The fragment crystallizable (Fc) region is a highly immunogenic factor capable of triggering an inflammatory response through its interaction with intraretinal Fc receptors [11]. This inflammatory reaction can be traced back to the Fc antibody portion seen in aflibercept and bevacizumab. This fragment is noticeably absent in the ranibizumab molecule [12]. In contrast, faricimab has a modified Fc region that has been specifically optimized to prevent binding with Fc-gamma receptors (FcγRs) and neonatal Fc receptors. This specification helps reduce inflammation and limits systemic exposure, thereby alleviating these concerns of paramount importance [11,12].

Factors associated with the manufacturing and formulation of the product that may incite IOI include the presence of bacterial endotoxins, impurities, or proteins from non-human sources [11,13]. In addition, delivery-related factors such as syringe agitation, silicone oil-induced protein aggregates in the syringe, freeze-thawing processes, as well as shipping and handling procedures may all lead to inflammation [11,14,15].

## Conclusions

We report three cases, two cases of mild vitritis without retinal vasculitis and one case of anterior uveitis associated with IVT faricimab, that responded well to topical steroid therapy. One patient had a prior history of IOI (mild vitritis) following IVT brolucizumab in the past. This may have heightened the susceptibility for an immune response due to anti-drug antibodies (ADAs) that led to the second episode of IOI following faricimab. On the other hand, the other two patients had no prior history of IOI or IVT brolucizumab. This disparity in prior drug exposure warrants further research into the role of possible immunological mechanisms underlying IOI and thereby help in its prevention.
